# Long Noncoding RNA-H19 Contributes to Atherosclerosis and Induces Ischemic Stroke via the Upregulation of Acid Phosphatase 5

**DOI:** 10.3389/fneur.2019.00032

**Published:** 2019-02-04

**Authors:** Yujing Huang, Liping Wang, Ying Mao, Guangxian Nan

**Affiliations:** Department of Neurology, China-Japan Union Hospital of Jilin University, Changchun, China

**Keywords:** atherosclerosis, ischemic stroke, long noncoding RNA-H19, acid phosphatase 5, differentially expressed genes

## Abstract

**Objective:** Atherosclerosis is closely associated with ischemic stroke, and long noncoding RNA-H19 (lncRNA-H19) might be a potential target for treating atherosclerosis. The present study aimed to investigate the function of lncRNA-H19 in atherosclerosis and to explore a novel therapeutic strategy for ischemic stroke.

**Methods:** Differentially expressed genes (DEGs) in atherosclerosis were screened by searching public database. In combination with the lncRNA-H19-knockout database, potential lncRNA-H19-mediated gene was retrieved and their relationship was identified. In order to assess the detailed regulatory mechanism of lncRNA-H19, we used a lentivirus packaging system to upregulate Acp5 (Acid phosphatase 5) expression in vascular smooth muscle cells (VSMC) and human umbilical vein endothelial cells (HUVECs). The expression of ACP5 was determined by Western Blot, and evaluations of cell proliferation and apoptosis were detected. An ischemic stroke mouse model was established. Atherosclerosis was measured by using plaque area size. The effects H19 on the expression of ACP5 were explored by the overexpression or silence of H19.

**Results:** H19 and ACP5 were associated with Acute Stroke Treatment (TOAST) subtypes of atherosclerotic patients. The target prediction program and dual-luciferase reporter confirmed ACP5 as a direct target of H19. Lentivirus-mediated H19-forced expression upregulated ACP5 protein levels, promoted cell proliferation and suppressed the apoptosis. The plaque area size was larger in ischemic models than controls. The overexpression or silence of H19 increased or reduced the plaque size. The overexpression or silence of H19 resulted in the expression or inhibition of ACP5.

**Conclusion:** IncRNA-H19 promoting ACP5 protein expression contributed to atherosclerosis and increased the risk of ischemic stroke.

## Introduction

Ischemic stroke has been prevalent in middle-aged and elderly people with the second highest rate of lethality in China. This malignant disease seriously affects people's health and living quality (1–4). Atherosclerotic cerebral infarction, the most common type in ischemic stroke, remains a chronic compensatory inflammatory reaction induced by the interaction of genetic and environmental factors (5, 6). Heritability for stroke is 16.1, 32.6, and 40.3% for small-vessel disease, cardioembolic and large-vessel disease, respectively (7). Localized intimal thickening is the basic lesion of atherosclerosis (8). Inflammation is responsible for the formation of atherosclerotic plaque, which is the leading cause of ischemic stroke, and intervention in inflammatory response will delay atherosclerotic plaque formation and rupture (9, 10). Additionally, lipoprotein-associated phospholipase A2 (LP-PLA2) is an inflammatory response marker associated with the formation of atherosclerotic plaque (11). Epidemiological studies have confirmed that smoking, drinking, diabetes, obesity, high-density lipoprotein, and low cholesterol are risk factors for cerebrovascular disease, especially for ischemic cerebrovascular disease (12–15). It is reported that atherosclerosis is the main cause of coronary heart disease, cerebral infarction and peripheral vascular disease (16).

Long non-coding RNAs (lncRNAs) are normally longer than 200 bp in length. Recently, lncRNAs have become the focus of genetics research due to its important role in numerous life activities such as dosage compensation effect, epigenetic regulation, cell cycle, and cell differentiation regulation (17–20). lncRNAs in plasma and serum have been considered as a new biomarker for the diagnosis and prognosis of cardiovascular disease (21). Evidence has been provided that lipid metabolism-associated lncRNAs are pertinent to the risk and severity of coronary artery disease (22).

However, there are still few studies on lncRNAs in atherosclerosis-induced ischemic stroke. Long non-coding RNA (lncRNA) H19 is located on human chromosome 11. In the central nervous system, H19 is highly expressed in glioblastoma tissue (23). LncRNA H19 has been reported to be overexpressed in atherosclerotic patients, and may be a potential target in the prevention of atherosclerosis (24). lncRNA-H19 polymorphism has been found to be associated with small vessel ischemic stroke susceptibility in the Chinese Han population and can be developed a potential biomarker for the diagnosis of ischemic stroke susceptibility (25). Elevated level of lncRNA H19 was detected in the blood of the patients with coronary artery disease patients, suggesting that H19 may be involved with atherosclerosis process and a new biomarker for the diagnosis of coronary artery disease (26, 27). H19 may be used as the target for the therapy of atherosclerosis by regulating WNT/beta-catenin signaling pathway in vascular smooth muscle cells (28). H19-miR130b pathway is associated with lipid metabolism and inflammation activities, also suggesting that H19 is a new target for atherosclerosis therapy (29). Thus, LncRNA H19 may an important cause of ischemic stroke.

In our present study, we screened atherosclerosis-related gene lncRNA-H19 and its target gene ACP5 by using bioinformatics technique. The experiments demonstrated that lentivirus-mediated stromal cell-derived factor-1 (SDF-1)-forced expression upregulated the level of ACP5, promoted the proliferation, and inhibited the apoptosis of arterial intimal cells, revealing lncRNA-H19-mediated atherosclerosis. H19 may be a new target for the treatment of ischemic stroke.

## Materials and Methods

### Participants

All procedures were approved by Human Research Ethical Committee of China-Japan Union Hospital of Jilin University (Changchun, China, approval No. 20150420). Eighty-five ischemic stroke patients who were hospitalized in China-Japan Union Hospital of Jilin University during May 2015–May 2017 years were checked by CT or MRI examination. Among them, 80 cases were confirmed as cerebral infarction and 5 cases as hemorrhagic infarction. Madison ultrasound system (Jeju, South Korea) with transducer frequency of 7.5 MHz was applied to determine plaque site and range as well as internal and middle membrane thickness (IMT). Two neurologists with more than 10-year clinical experience to segment the plaques in ultrasound videos of the common carotid artery (CCA) based on frame normalization. They manually delineated the borders between plaque and artery wall. The plaque area was calculated by using the software MATLAB. Carotid atherosclerosis was identified by IMT ≥1.0 mm. According to the Acute Stroke Treatment (TOAST) Classification, ischemic strokes were classified into (1) large-artery atherosclerosis (LAA), (2) small vessel occlusion (SVO), (3) cardioembolism (CE), (4) stroke of other determined etiology (OD), and (5) stroke of undetermined etiology (UD) (30). All patients were informed and signed a written consent form and premedicated with intravenous 1,000 U heparin to prevent thrombosis. Meanwhile, 85 healthy subjects were selected as a control group. The significant differences for age, sex, smoking, and drinking cases were analyzed between the atherosclerotic group and the control group. Two-mL blood was obtained from each subject and serum was isolated via centrifugation at 3000 × *g* for 10 min. The serum levels of LDL-C (ab91115), HDL-C (ab204717), TC (ab14273), TG (ab65336), and hs-CRP (ab99995) were analyzed by using the corresponding kits from Abcam (Shanghai Branch Office, China).

### Data Screening and Analysis

“Carotid artery atheroma” was retrieved as the key word in Gene Expression Omnibus (GEO) and GSE43292 data set was obtained. The data set contained two subgroups, the atherosclerotic plaque and the intact tissue at a greater distance from the plaque in hypertensive patients. Subsequently, GSE76741 data were acquired from GEO with “H19” as a keyword, setting species as “Homo sapiens.” The analysis of variance was performed using the Limma package in R language and significant difference was defined as *P* < 0.05 and |log fold change (FC)| > 1. Gene Ontology (GO) and Kyoto Encyclopedia of Genes and Genomes (KEGG) enrichment analysis was carried out via DAVID (https://david.ncifcrf.gov/) (31).

### Cell Culture

Vascular smooth muscle cells (VSMC) and human umbilical vein endothelial cells (HUVECs) were purchased from the Institute of Biochemistry Cell Biology (Shanghai, China). The cells were maintained in a humidified incubator with 95% air and 5% CO_2_ at 37°C, and cultured in Dulbecco's minimum essential medium (DMEM) (Hyclone, South Logan, UT, USA) containing with 1% penicillin/streptomycin (100 U/mL/100 mg/mL) (Beyotime, Beijing, China) and 10% fetal bovine serum (FBS) (Gibco, Grand Island, NY, USA).

### Vector Construction

All plasmids were purchased from Shanghai Sangon Biotech Company (Shanghai, China). Before twenty-four-hour transfection, the cells were inoculated in 6-well plates and cell transfection began to perform in line with the instruction of lipofectamine 2000 (11668-019, Invitrogen, New York, CA, USA) when cell density reached 50% confluency. A volume of 250 μL serum-free Opti-MEM (51985042, Gibco, Gaitherburg, MD, USA) was used to dilute 100 pmol plasmids with a final concentration of 50 nM and 5 μL lipofectamine 2000, fully mixed and incubated for 5 min at room temperature. The above two diluents were blended, cultured for 20 min at room temperature, incubated at 37°C in 5% CO_2_ for 6–8 h with cell culture medium, and then incubated for 24–48 h with complete medium. The cells were collected for subsequent experiment.

### Western Blot Analysis

Anti-ACP5 antibody (Cat.no. ab185716), anti-GAPDH antibody (Cat. no. ab226408) and HRP Goat Anti-Mouse (IgG) secondary antibody (ab97023) were purchased from Abcam. The myeloid tissues were washed in 20 mM PBS buffer (pH 7.0), ground, added with cell lysate containing protease inhibitor Cocktail (Roche, Indianapolis, IN, USA), vibrated for 5 min at 4°C, and centrifuged at 14,000 rpm for 10 min at 4°C. The supernatant was collected to extract protein through Qproteome Mammalian Protein Prer kit (QIAGEN, GmbH, Germany) and preserved at −20°C. A total of 50 ug protein was processed by sodium dodecyl sulfate polyacrylamide gel electrophoresis (SDS-PAGE) and transferred to nitrocellulose (NC) membrane. The NC membrane was cultured in buffer solution (10 mmol/L Tris-HCl (pH 8.0), 150 mmol/L NaCl, and 0.05% Tween-20), blocked in 5% skim milk for 1 h, and then incubated overnight with first antibody at 4°C, followed by horseradish peroxidase (HRP)-bound second antibody (1:5,000; Beijing Zhongshan biotechnology Co., Ltd., Beijing, China). The product was exposed to enhanced chemiluminescence reagent (Amersham Biosciences, Fairfield, CT, USA), and antigen-antibody complex was visualized via X-ray film. Western blot analysis was performed using Image J software (National Institutes of Health). Each sample was normalized according to glyceraldehyde phosphate dehydrogenase (GAPDH) parameters. The experiment was repeated in triplicates.

### 3-(4,5-dimethyl-2-thiazolyl)-2,5-diphenyl-2-H-tetrazolium bromide (MTT) Assay

The cells were treated with 0.25% trypsin, centrifuged and resuspended in DMEM. Then, the cells were inoculated into a 24-well flat-bottomed plate at 3 × 10^4^ cells/well and cultured in 5% CO_2_ at 37°C for 0, 2, 4, and 6 days separately. Each well was added with 20 μL MTT (5 mg/mL) for 4 h of incubation. After the removal of supernatant, each well was added with 200-μL dimethyl sulfoxide (DMSO), and incubated or oscillated for 10 min at 37°C until the blue-violet crystal dissolved completely. The absorbance was measured at 570 nm by a microplate reader and cell growth curve was drawn.

### Flow Cytometry

The apoptosis rate was determined by Annexin V-7-ADD (Roche Corp., Basel, Switzerland). Briefly, after 48-h transfection, cells were collected, washed twice with precooled PBS, resuspended in 200 μL of binding buffer and incubated in dark for 20 min with 20 μL Annexin-V-R-PE in a manner of ice bathing, followed by adding 10 μL AAD. Subsequently, flow cytometry was conducted. DMSO was used as the negative control.

### Dual-Luciferase Reporter Assay

The full-length ACP5 was amplified by polymerase chain reaction (PCR) from genomic DNA and cloned into the XhaI and EcoRI sites of a pGL3-BS vector (Promega Corporation, Madison, WI, USA). The mutant construct of ACP5 was established using a QuikChange mutagenesis kit (Stratagene; Agilent Technologies, Inc., Santa Clara, CA, USA). pGL3 vector contains a high-copy-number prokaryotic origin of replication used in *E. coli*, an ampicillin-resistance gene for selection, and f1 ori of replication for single-stranded DNA production. Restriction sites for DNA insertion are located upstream and downstream of the luciferase gene (**Figure 7A**). Co-transfection of reporter vectors and TUC338 siRNA or NC was performed using lipofectamine 2000 (Thermo Fisher Scientifc, Inc., San Jose, CA, USA). TUC338 (transcribed ultra-conserved region 338) is a non-coding RNA. TUC338 silence may reduce the growth rate of VSMC and HUVECs (32). After 48 h, dual-luciferase activity was examined using a Dual-Luciferase reporter assay system (Promega Corporation, Madison, WI, USA).

#### Establishment of Ischemic Stroke Model

C57/BL6 mice (weighting 18–22 g, 4 weeks) were purchased from Laboratory Animal Center, CAS (Shanghai, China), and were housed in a 22.5 ± 0.5°C room with free access to food and water under 12:12-h light/dark. Surgical procedures were approved by Animal Care and Use Committee of China-Japan Union Hospital of Jilin University (Changchun, China). For the establishment of ischemic stroke model, the right somatosensory cortex was occluded from distal branches of the middle cerebral artery. The mice were anesthetized by 2% isoflurane. A 10-mm was prepared on the midway between right eye and ear. Three distal branches of middle cerebral artery were permanently ligated with a 4.0 silk suture for a 10-min period, and separated by a 20-min rest period. The mice were ligated for 5 times as repetitive mild hypoxia-ischemia as transient hypoxia-ischemia (tHI). Subsequently, the mice received 15-min hypoxia (7.5% O2 and 92.5% N2) daily for 3 days. For tHI, the mice were exposed to hypoxia (7.5% O2 and 92.5% N2) for 30 min.

### The Following Information Was Added in Result Section

A mouse model with ischemic stroke was established via the occlusion of distal branches of the right MCA after 10-min ligations for 5 times. This ischemic treatment could damage the right somatosensory cortex of mice (33). The measurement of periprocedural cerebral blood flow was performed using laser-Doppler flowmetry (PF2B; Perimed, Stockholm, USA). The cerebral blood flow was reduced in rmHI group by more than 40% in rHI group.

#### H19 Overexpression and Silence in Ischemic Stroke Models

H19 was overexpressed or silenced using an adeno-associated viral (AAV) plasmid. Briefly, a plasmid with H19 (AAV-H19) or a short hairpin RNA targeting H19 (AAV-shRNA) was synthesized from TaKaRa (Dalian, China). Five-microliter plasmids were injected into the right dorsal and ventral hippocampus of mouse models at 0.2 μL/min.

### Measurement of lncRNA-H19

Total RNA was extracted from cells or aortic tissue (homogenized) using TRIzol reagent (Invitrogen Corp., Carlsbad, CA, United States), and then reverse-transcribed using GeneAmp Kit (Perkin Elmer, Norwalk, CT, USA). lncRNA H19 level was measured using real-time qRT-PCR on MX3000 instrument (Agilent, La Jolla, CA, USA). PCR was performed in a total reaction volume of 10 μl, including 5 μL 2 × PCR master mix (SYBR Premix Ex Taq), 0.5 μL of PCR Forward Primer (Table S4, 10 μM), 2 μL of cDNA, and diluted to 20 μL ddH_2_O. The quantitative real-time RT-PCR reaction was set at an initial denaturation step of 3 min at 94°C; and 94°C 10 s, 58°C 40 s, 94°C 10 s in 45 cycles, with a final step from 58 to 94°C. GAPDH was used as a control to calculate relative level of lncRNA H19.

### Statistical Analysis

All statistical analysis was done using Graphpad Prism 5.0 software and all measurement data were expressed as mean ± standard deviations (SD). One-way analysis of variance (ANOVA) was used for the comparison between two groups. *p* < 0.05 indicates statistical significance.

## Results

### Baseline Demographic Characteristics

There was no significant difference in age, sex, smoking and drinking between the atherosclerotic group and the control group (*P* > 0.05, Table 1), but the cases of patients with hypertension and diabetes in the atherosclerotic group was significantly higher than that of the control group (*P* < 0.05, Table 1). The levels of hs-CRP, LDL, TG, and TC in peripheral blood of atherosclerotic group patients were higher than those of the control group (*P* < 0.05, Table 1), while the HDL content was significantly lower than that of the control group (*P* < 0.01, Table 1).

**Table 1:** Baseline demographic characteristics between two groups.

**Parameters**	**Atherosclerotic group (*n* = 85)**	**Control group (*n* = 85)**	**X2/*t* values**	***P* values**
Age	58.90 ± 13.54	56.94 ± 10.45	0.937	0.350
Sex, male cases (%)	47 (55.29)	47 (55.29)	0.000	1.000
Smoking, cases (%)	35 (41.18)	30 (35.29)	0.623	0.430
Drinking, cases (%)	21 (24.71)	23 (27.06)	0.123	0.726
Hypertension, cases (%)	52 (61.18)	12 (14.12)	38.358	0.000
Diabetes, cases (%)	26 (30.59)	8 (9.41)	11.912	0.001
**TOAST CLASSIFICATION CASES (%)**				
LAA	34 (40.00)		
SVO	16 (18.82)		
CE	18 (21.18)		
OD	3 (3.53)		
UD	14 (16.47)		
LDL (mmol/L)	2.81 ± 0.53	2.46 ± 0.56	3.849	0.000
HDL (mmol/L)	1.14 ± 0.24	1.28 ± 0.26	−3.488	0.001
TC (mmol/L)	5.20 ± 0.88	4.47 ± 0.86	5.220	0.000

*According to the TOAST classification, ischemic strokes were classified into (1) large-artery atherosclerosis (LAA), (2) small vessel occlusion (SVO), (3) cardioembolism (CE), (4) stroke of other determined etiology (OD), and (5) stroke of undetermined etiology (UD)*.

### Analysis of Differentially Expressed Genes (DEGs)

Subseiquently, 135 DEGs of atherosclerosis were found in GSE43292 database, among which 123 DEGs have been annotated (Table S1).

**Figure 1:**
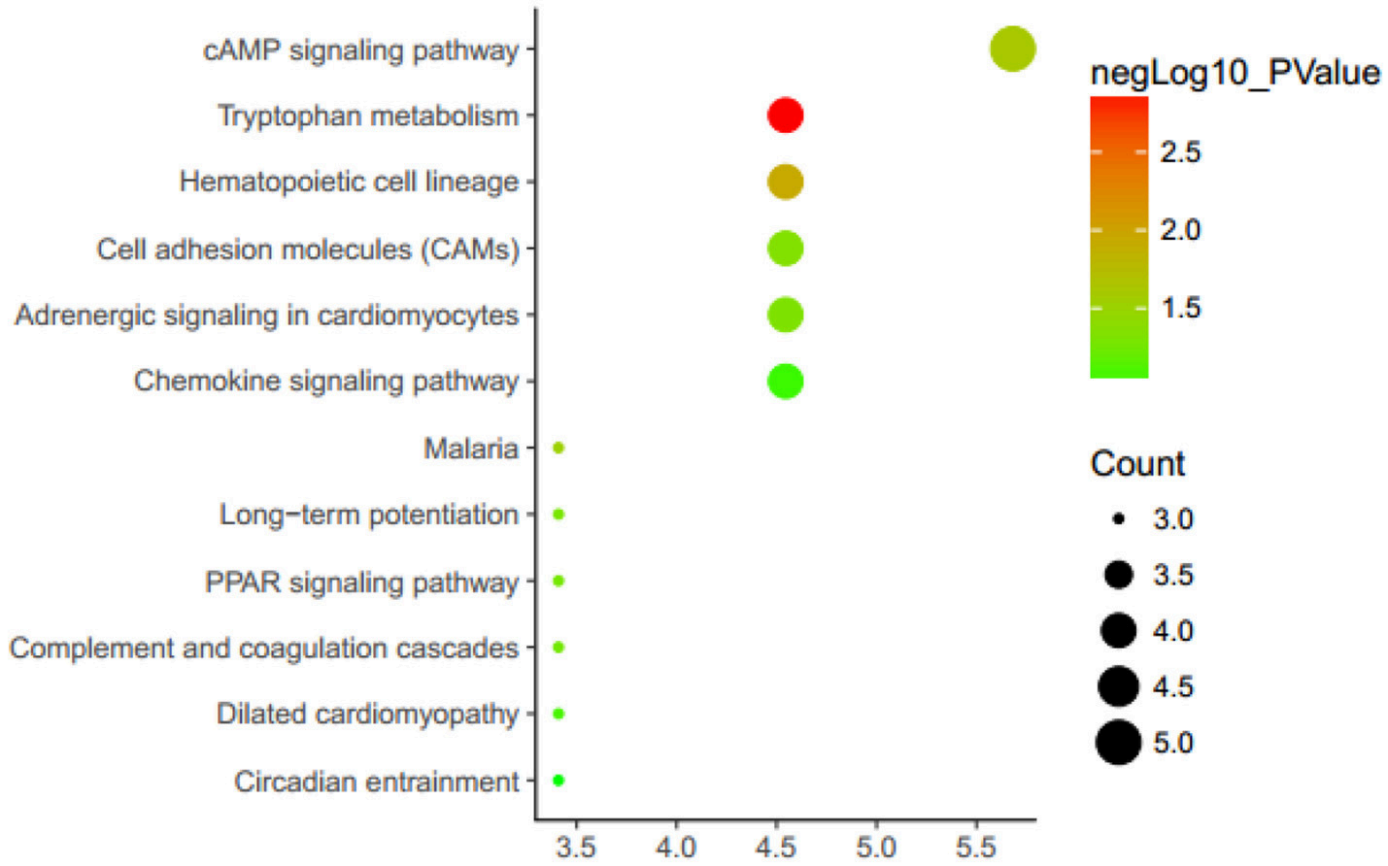
Enrichment analysis KEGG metabolic pathways. KEGG, Kyoto Encyclopedia of Genes and Genomes. Genes are associated with up/down expression levels (green/yellow/red coloring).

To further understand the function of these DEGs, enrichment analysis was performed using the DAVID (Figure 1 and Table S2). In the enrichment of metabolic pathways, these DEGs were mainly enriched in “cAMP signaling pathway” and “hematopoietic cell lineage,” etc. A recent study has revealed that cAMP signaling pathway is correlated with atherosclerosis (34), and hematopoietic cell lineage also has a certain association with the onset of atherosclerosis. It suggests that these DEGs are most likely associated with atherosclerosis.

### Retrieval of Atherosclerosis-Related lncRNAs

With “atherosclerosis” as the key word retrieved in NONCODE, H19, ANRIL, and CDKN2B-AS1 were found to be involved in atherosclerosis onset (Table 2). A lot of literatures show that lncRNA-ANRIL (35) and CDKN2B-AS1 (36, 37) are closely related to the occurrence of atherosclerosis. Moreover, in the GEO, there is already relevant data on the effects of ANRIL on the downstream genes in atherosclerosis (38). In order to understand the association between lncRNA and atherosclerosis, we investigated the functional role of lncRNA-H19 in the development of atherosclerosis.

**Table 2:** Atherosclerosis-related lncRNAs.

**NONCODE ID**	**Gene symbol**	**Disease name**	**Source database**	**Source PMID**
NONHSAG007409.2	H19	Atherosclerosis	LncRNADisease	21954592
NONHSAG101229.2	ANRIL	Atherosclerosis	LncRNADisease	23861667
NONHSAG101229.2	ANRIL	Atherosclerosis	LncRNADisease	23813974
NONHSAG101229.2	CDKN2B-AS1	Atherosclerosis	LncRNADisease	19592466
NONHSAG101229.2	CDKN2B-AS1	Atherosclerosis	LncRNADisease	20637465
NONHSAG101229.2	CDKN2B-AS1	Atherosclerosis	LncRNADisease	20956613
NONHSAG101229.2	CDKN2B-AS1	Atherosclerosis	LncRNADisease	20056914
NONHSAG101229.2	CDKN2B-AS1	Atherosclerosis	LncRNADisease	21550161
NONHSAG101229.2	CDKN2B-AS1	Atherosclerosis	LncRNADisease	23791884
NONHSAG101229.2	CDKN2B-AS1	Atherosclerosis	LncRNADisease	19343170
NONHSAG101229.2	CDKN2B-AS1	Atherosclerosis	LncRNADisease	21151960
NONHSAG101229.2	CDKN2B-AS1	Atherosclerosis	LncRNADisease	22178423
NONHSAG101229.2	CDKN2B-AS1	Atherosclerosis	MNDR	22178423

### Chip Data Analysis After lncRNA-H19 Knockout

**Figure 2:**
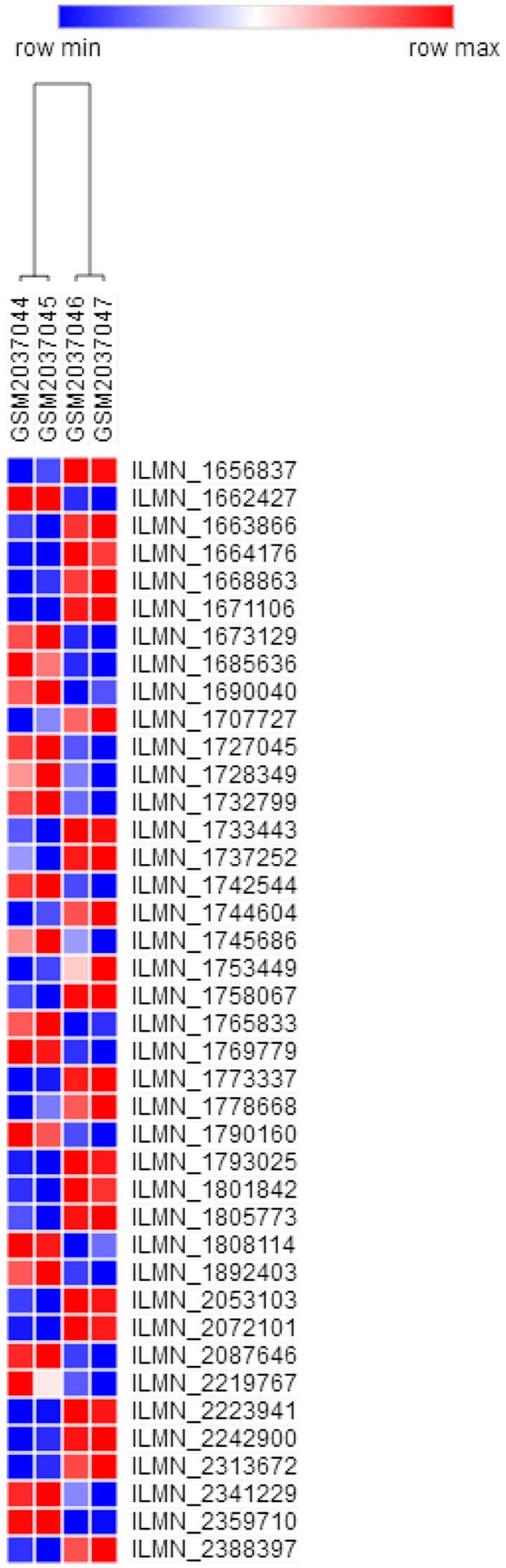
Heat map of DEGs (top 40 in fold change). Colors ranging from blue to red represent the DEGs' average fold change among the subjects. DEGs, Differentially expressed genes.

GSE76741 database contains control group and H19-knockout group, between which there were 308 annotated DEGs (158 and 150 downregulated and upregulated genes). We speculated that the changes in the expression of these DEGs were most likely due to H19 knockout (Table S3 and Figure 2).

### Venn Diagram Analysis of DEGs

**Figure 3:**
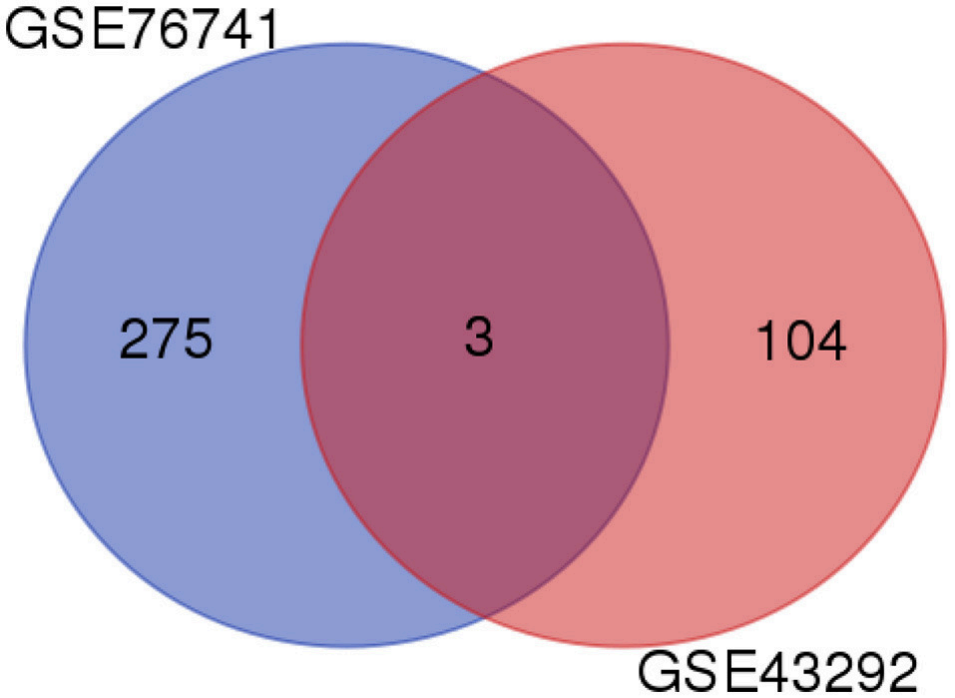
Venn diagram analysis of DEGs. Venn diagram of DEGs is presented in the pairwise melanic-typical comparisons. DEGs, Differentially expressed genes.

Through the above data analysis, 308 potential target genes of lncRNA-H19 were discovered, but these target genes were not necessarily associated with atherosclerosis. Therefore, Venn diagram analysis was subsequently performed to identify the correlation between H19 target genes and atherosclerosis-related genes (Figure 3). Finally, ACP5, MRAP2, and MME were found to show associations with both atherosclerosis and lncRNA-H19.

### ACP5 Was Identified as a Direct Target Gene of H19

Next, we retrieved the nucleotide sequences of H19, ACP5, MRAP2, and MME via the lncRNA database and NCBI database. Based on the nucleotide sequences, the targeted binding between H19 and the remaining three genes were predicted. The results showed that there may be a direct regulatory relationship between H19 and ACP5 (Table 3).

**Table 3:** ACP5 was identified as a direct target gene of H19.

**Query**	**Length query**	**Target**	**Length target**	**dG**	**ndG**	**Start position query**	**End position query**	**Start position target**	**End position target**
H19	2362	ACP5	978	−97.8	−0.102	286	1263	1	978

#### H19 and ACP5 Expressions Were Elevated in Patients With Atherosclerosis

**Figure 4:**
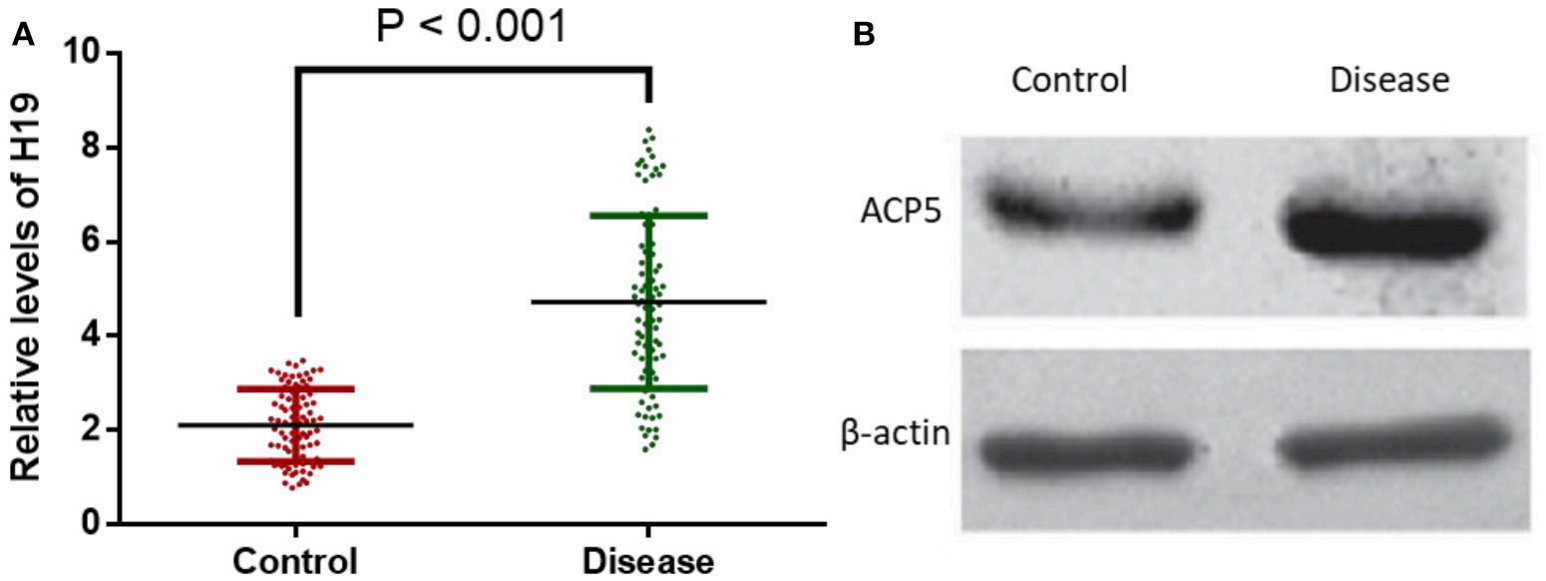
H19 and ACP5 expressions were elevated in patients with atherosclerosis. **(A)**, relative levels of H19. **(B)**, relative protein level of ACP5. Control, healthy subjects; Disease, the patients with atherosclerosis.

Subsequently, the expressions of H19 and ACP5 in blood serum of atherosclerotic patients and healthy population were determined. The results showed that H19 and ACP5 expressions were significantly increased in atherosclerotic patients, suggesting the positive regulatory effect of H19 (Figure 4A) on ACP5 expression (Figure 4B).

#### H19 and ACP5 Were Associated With TOAST Subtypes of Atherosclerotic Patients

**Figure 5:**
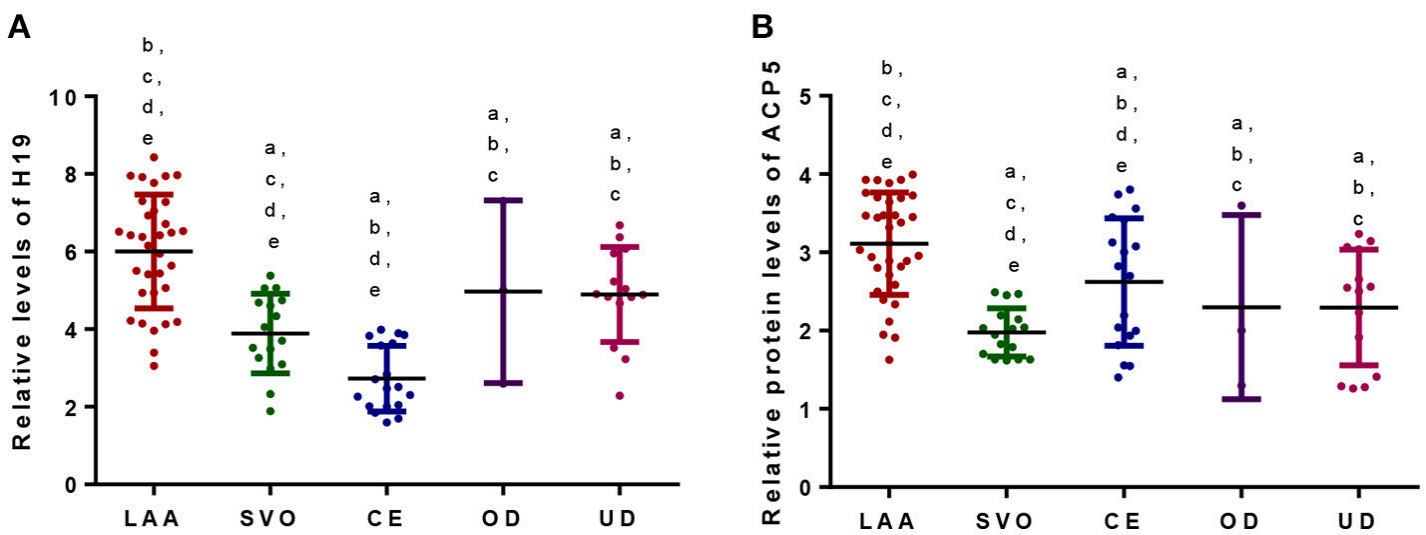
**(A)** Relative levels of H19 among different TOAST subtypes of atherosclerotic patients. **(B)** Relative levels of ACP5 among different TOAST subtypes of atherosclerotic patients. According to the TOAST classification, ischemic strokes were classified into (1) large-artery atherosclerosis (LAA), (2) small vessel occlusion (SVO), (3) cardioembolism (CE), (4) stroke of other determined etiology (OD), and (5) stroke of undetermined etiology (UD). ^a^*P* < 0.05 vs. a LAA group; ^b^*P* < 0.05 vs. a SVO group; ^c^*P* < 0.05 vs. a CE group; ^d^*P* < 0.05 vs. a OD group and ^e^*P* < 0.05 vs. an UD group.

Patients were assigned into five subtypes according to TOAST classification (Table 2). The levels of H19 (Figure 5A) and ACP5 (Figure 5B) were highest in the patients with LAA (*P* < 0.05). Patients with CE had the lowest levels of H19 (Figure 5A) and ACP5 (Figure 5B) (*P* < 0.05). However, no difference was found between OD and UD groups (Figure 5, *P* > 0.05).

### lncRNA-H19 Overexpression Inhibits Cell Apoptosis

**Figure 6:**
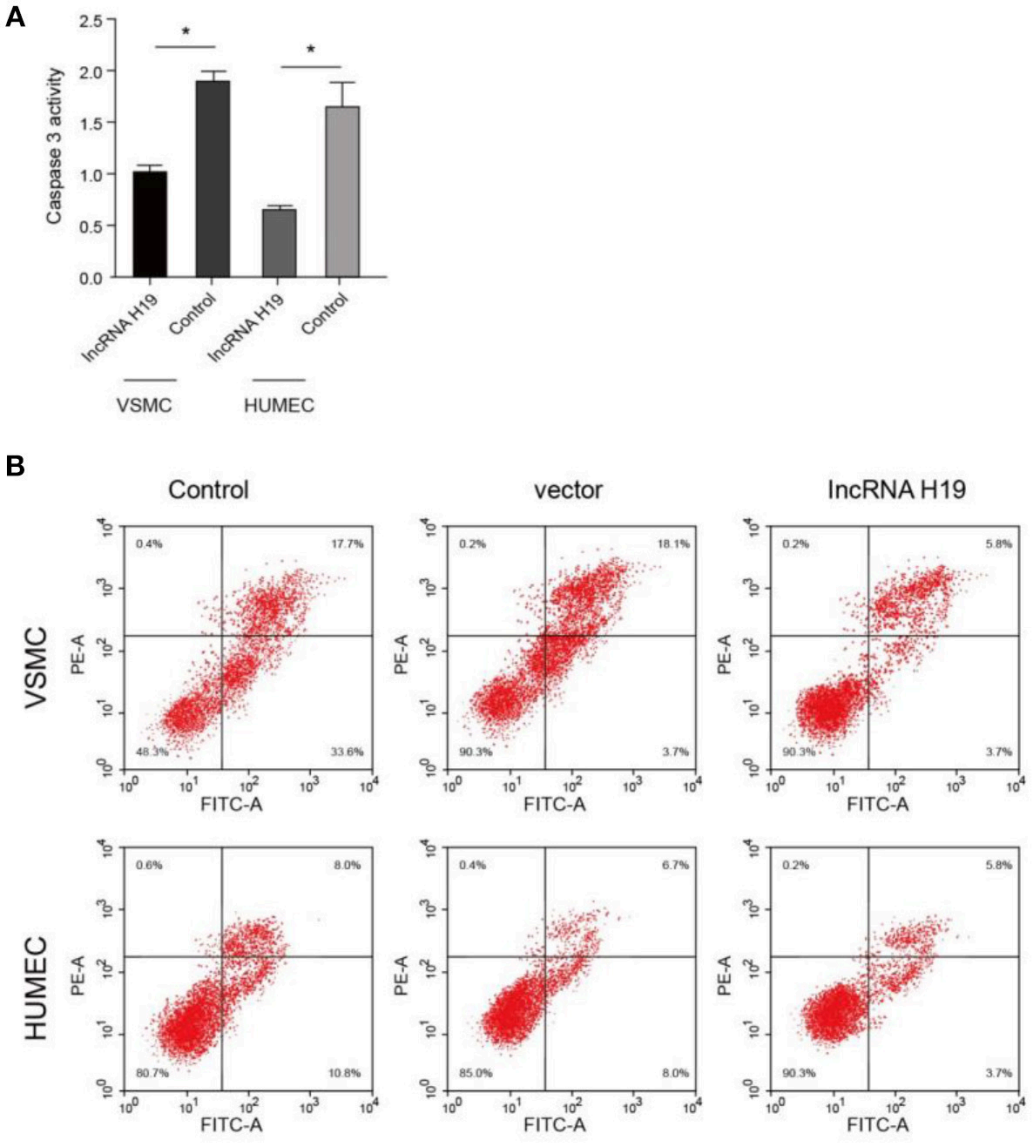
LncRNA-H19 overexpression inhibited cell apoptosis. **(A)**, caspases 3 activity in VSMC and HUMEC. **(B)**, apoptosis analysis in Vascular smooth muscle cells (VSMC) and human umbilical vein endothelial cells (HUVECs). ^*^p < 0.001 vs. the control group.

Simultaneously, an evaluation of cell apoptosis was detected. Our results revealed that caspase3 (apoptotic marker protein) was significantly downregulated in lncRNA-H19-overexpressed cell lines (Figure 6A). Supportably, flow cytometry also showed declined cell apoptosis (Figure 6B). These results suggest that lncRNA-H19 overexpression can inhibit cell apoptosis.

#### lncRNA-H19 Positively Regulates ACP5 Expression at the Post-transcript Level

**Figure 7:**
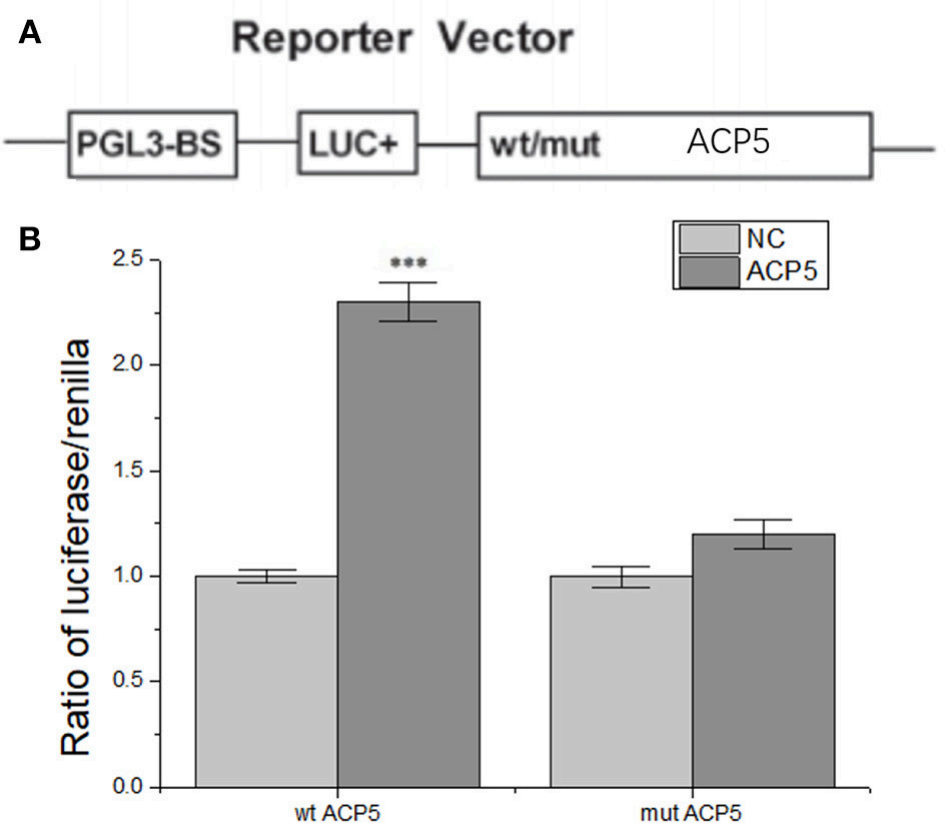
lncRNA-H19 positively regulates ACP5 expression at the post-transcript level. **(A)**, structural elements of the pGL3 luciferase reporter vectors. **(B)**, ratio of luciferase/renilla. ^***^p < 0.001 vs. the NC group.

To further understand the regulatory relationship between H19 and ACP5, dual-luciferase reporter system was constructed. The results showed that the activity of fluorescent protein was significantly enhanced after co-transformation of ACP5 mRNA/pGL3-BS and lncRNA-H19 (Figure 7), indicating that lncRNA-H19 upregulates the content of ACP5 protein.

#### H19 Regulated the Expression of ACP5 in Ischemic Stroke Models

**Figure 8:**
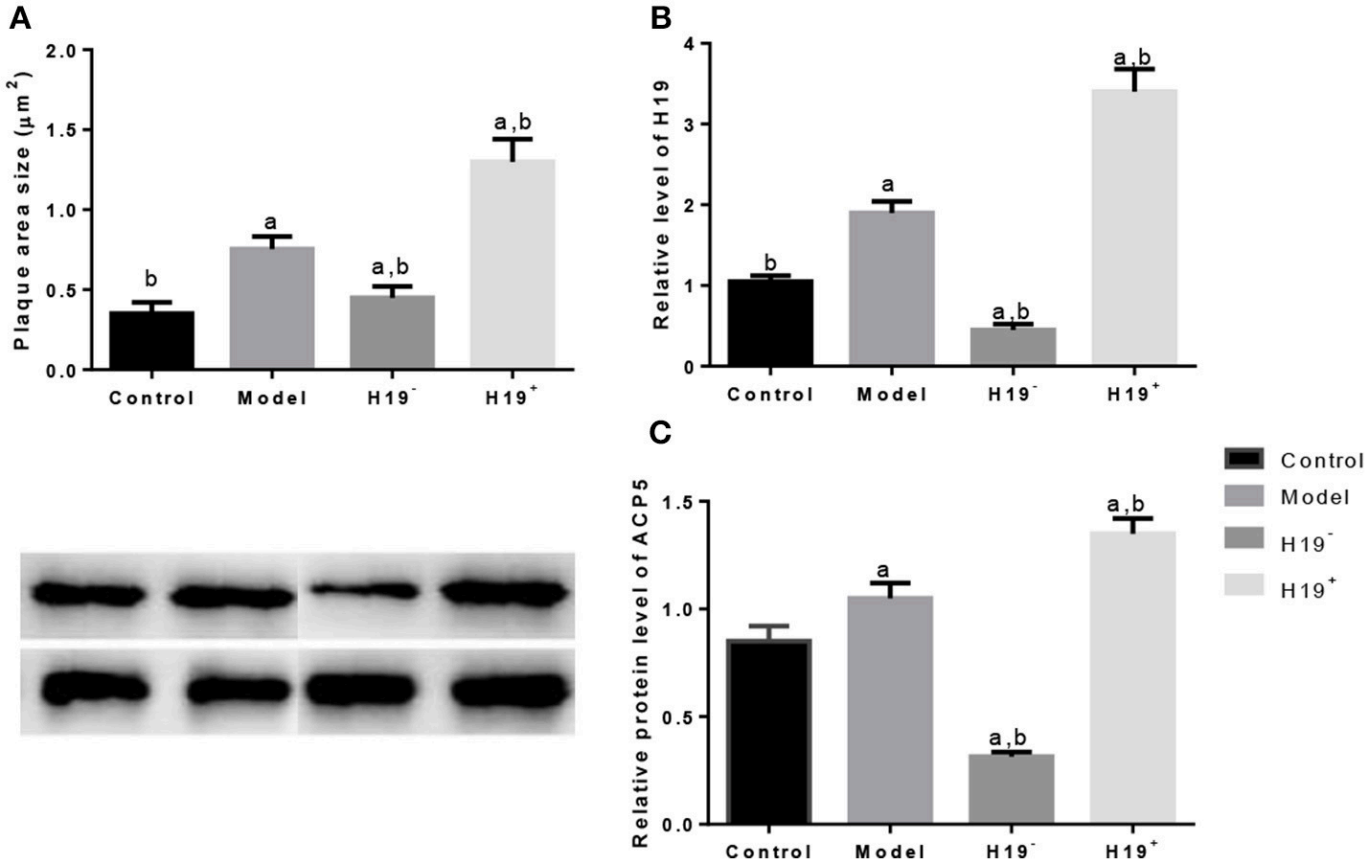
H19 induces ACP5 expression in ischemic stroke models. **(A)**, plaques area size; **(B)**, the relative level of H19; **(C)**, Protein levels of ACP5. Data represent mean ± S.D.H19^+^, a vector AAV harboring H19; H19^−^, a vector AAV harboring a short hairpin RNA targeting H19. ^a^*P* < 0.05 vs. a control group and ^b^*P* < 0.05 vs. a model group.

Atherosclerosis was measured according to the plaque area determined by staining of the aortic arch with oil red-O solution. The area size of atherosclerosis was significantly larger in model group than in control group (Figure 8A). H19 overexpression increased the plaque area size while the silence reduced the size significantly (Figure 8A). Relative level of H19 was higher in model group than in control group (Figure 8B). H19 vector increased the H19 level while the silence of H19 reduced the level significantly (Figure 8B). Similarly, ACP5 level was higher in model group than in control group (Figure 8C). H19 vector increased the ACP5 level while the silence of H19 reduced the ACP5 level significantly (Figure 8C). The results suggested H19 regulated the expression of ACP5.

## Discussion

Coronary artery disease and ischemic stroke arising from atherosclerosis are major causes of morbidity and death worldwide (39). Atherosclerosis can lead to cerebral artery stenosis or occlusion, successive ischemic-hypoxic necrosis, and finally ischemic stroke (40). lncRNA plays an important role in a variety of biological processes such as cell differentiation and proliferation, gene transcription and translation. As the main pathological factor, atherosclerosis is induced by endothelial injury and activation, leading to infiltration and proliferation of VSMC, leukocytes, and other inflammatory corpuscles (41). Recently, lncRNA has become a major research focus for the treatment of ischemic stroke. For instance, lncRNA MALAT1 inhibits human endothelial vascular cell proliferation (42). In this study, we analyzed relevant data and screened the differentially expressed genes in atherosclerotic diseases. Previous evidence showed that lncRNA-H19 was highly expressed in neointima after injury and in atherosclerotic patients, but the mechanism of lncRNA-H19 in atherosclerosis was not elucidated completely. Therefore, this investigation was primarily focused on lncRNA-H19 with the expectation to explore a novel therapeutic target for atherosclerosis.

lncRNA-H19 is an important regulator in the progression of atherosclerosis, which leads to ischemic stroke. H19 can cause neuroinflammation by affecting histone deacetylase 1-dependent M1 microglial polarization and may be an important target for the therapy of ischemic stroke (43). On the other hand, acid phosphatase can promote lipid synthesis (44), which causes the risk of ischemic stroke (45). The present study demonstrated that lncRNA-H19 promoting ACP5 protein expression contributed to atherosclerosis and increases the risk of ischemic stroke.

ACP5 encodes tartrate-resistant acid phosphatase (TRAP) that is a metalloenzyme expressed in activated osteoclasts and macrophages, which has recently gained traction as a driving factor for metastasis and is significantly associated with cancer progression (46, 47). Overexpression of TRAP increases anchorage-independent and anchorage-dependent cell growth and proliferation and promotes cell migration and invasion (47). It has also demonstrated that overexpression of ACP5 is associated with lung adenocarcinoma progression and may be a potential prognostic biomarker for lung adenocarcinoma (48). Elevated expression of ACP5 in hepatocellular carcinoma (HCC) tissues was found to be associated with microvascular infiltration, poor differentiation, high lymph node metastasis, and poor survival (49). Therefore, we speculate that ACP5 may cause atherosclerosis by affecting the proliferation and apoptosis of endothelial vascular cells. In the present study, the speculation was identified by significantly increased proliferation and decreased apoptosis in VSMC and HUVECs.

How is the expression of ACP5 affected? lncRNAs mediates cell proliferation, migration, and invasion by regulating the expression of downstream signaling molecules. For example, lncRNA-FTH1P3 promotes melanoma cell proliferation and invasion by targeting miR-224-5p (50); lncRNA-LINC00673 contributes to HCC progression and metastasis via negative regulation of miR-205 (51); lncRNA-NEAT1 up-regulates E2F3 expression and subsequently promotes the progression of non-small cell lung cancer (52). Remarkably, Wang discovered that lncRNA-H19 inhibited thyroid cancer cell activity, migration and invasion by down-regulating insulin receptor substrate 1 in SW579 and TPC-1 cells (53). Based on that, the current study identified the targeting relationship between lncRNA-H19 and ACP5 using the target prediction system and dual-luciferase reporter assay. Ischemic animal model also demonstrated that the plaque area size was larger in ischemic models than controls. The overexpression or silence of H19 increased or reduced the plaque size. The overexpression or silence of H19 resulted in the expression or inhibition of ACP5.

Collectively, all of the above findings validated that lncRNA-H19 regulates ACP5 expression at the post-transcriptional level by binding ACP5, and further affecting arterial endothelial vascular cell proliferation and apoptosis, ultimately resulting in atherosclerosis and ischemic stroke.

## Conclusion

To sum up, lncRNA-H19 is most likely associated with the occurrence of atherosclerosis. lncRNA-H19 induced the occurrence of atherosclerosis through positive regulation of ACP5 protein, leading to the emergence of ischemic stroke.

## Author Contributions

GN and YH conceived the experiments, read, and revised the manuscript. LW and YM wrote the manuscript, designed, and performed the experiments. All authors read and approved the final manuscript.

### Conflict of Interest Statement

The authors declare that the research was conducted in the absence of any commercial or financial relationships that could be construed as a potential conflict of interest.
