# Supplementary material for: Long Noncoding RNA-H19 Contributes to Atherosclerosis and Induces Ischemic Stroke via the Upregulation of Acid Phosphatase 5
Source: Front Neurol. 2019 Feb 4;10:32. doi: 10.3389/fneur.2019.00032 (PMC6369351; doi:10.3389/fneur.2019.00032)
Supplement: Supplementary file 4 [file Table_4.DOCX]

Table S4 Primers used in the present study

| Gene | | | 5’------------------------3’ |
| --- | --- | --- | --- |
| lncRNA-H19 | human | Forward | gcatgctccagagggaatc |
|  |  | Reverse | cacgtccaccggacctggc |
|  | Mouse | Forward | ggaatgttgaaggactgagg |
|  |  | Reverse | tctggggtcgaacccttccc |
| GAPDH | human | Forward | actacatggtttacatgttc |
|  |  | Reverse | tctccatggtggtgaagac |
|  | Mouse | Forward | tggtgaaggtcggtgtgaac |
|  |  | Reverse | gcaacaatctccactttgc |
